# Self-Reported Impact of the COVID-19 Pandemic on Nutrition and Physical Activity Behaviour in Dutch Older Adults Living Independently

**DOI:** 10.3390/nu12123708

**Published:** 2020-11-30

**Authors:** Marjolein Visser, Laura A. Schaap, Hanneke A. H. Wijnhoven

**Affiliations:** Department of Health Sciences, Faculty of Science, Amsterdsam Public Health Research Institute, Vrije Universiteit Amsterdam, De Boelelaan 1085, 1081 HV Amsterdam, The Netherlands; laura.schaap@vu.nl (L.A.S.); hanneke.wijnhoven@vu.nl (H.A.H.W.)

**Keywords:** coronavirus, malnutrition, undernutrition, obesity, physical activity, exercise, aged, quarantine, risk factors

## Abstract

The aim was to explore the self-reported impact of the COVID-19 pandemic on nutrition and physical activity behaviour in Dutch older adults and to identify subgroups most susceptible to this impact. Participants (N = 1119, aged 62–98 y, 52.8% female) of the Longitudinal Aging Study Amsterdam living independently completed a COVID-19 questionnaire. Questions on diagnosis, quarantine and hospitalization were asked, as well as impact of the pandemic on ten nutrition and physical activity behaviours. Associations of pre-COVID-19 assessed characteristics (age, sex, region, household composition, self-rated health, BMI, physical activity, functional limitations) with reported impact were tested using logistic regression analyses. About half of the sample (48.3–54.3%) reported a decrease in physical activity and exercise due to the pandemic. An impact on nutritional behaviour predisposing to overnutrition (e.g., snacking more) was reported by 20.3–32.4%. In contrast, 6.9–15.1% reported an impact on behaviour predisposing to undernutrition (e.g., skipping warm meals). Those who had been in quarantine (*n* = 123) more often reported a negative impact. Subgroups with higher risk of impact could be identified. This study shows a negative impact of the COVID-19 pandemic on nutrition and physical activity behaviour of many older adults, which may increase their risk of malnutrition, frailty, sarcopenia and disability.

## 1. Introduction

The COVID-19 pandemic and the public health measures related to COVID-19, such as social distancing, will likely have an impact on lifestyle behaviour. A clear need for studies examining this impact has been expressed [1,2], as this information is needed to develop public health actions and clinical interventions to reduce the negative impact of COVID-19 on lifestyle and optimize population health. Recent studies show that the COVID-19 pandemic is often associated with eating more, more snacking behaviour, more alcohol consumption but less binge drinking, more sedentary behaviour and body weight gain in adults [3,4,5,6,7]. Reported findings on the COVID-19 pandemic impact on physical activity are mixed, with studies reporting an increase [6] as well as a decrease [4] in physical activity levels or suggesting a differential change according to pre-COVID-19 physical activity levels [4,8].

It is hypothesized that older adults are more susceptible to a negative impact of the COVID-19 pandemic on lifestyle as compared to younger adults [9,10,11,12]. Social isolation as a result of public health measures such as social distancing and limiting social gatherings may decrease physical activity and decrease food intake due to lower food availability. Older adults may avoid grocery shopping due to fear of infection [3] or experience a reduction in support with shopping and cooking, which may negatively impact the energy content and variability of the diet. Furthermore, appetite might be reduced due to a lower physical activity level, eating alone and due to anxiety and stress from being at high-risk for COVID-19 complications. COVID-19-related behavioural changes have been hypothesized to subsequently contribute to an increased risk of malnutrition, sarcopenia and disability [13]. Furthermore, the COVID-19 infection itself may severely impact nutrition status of older adults [14]. Indeed, the prevalence of malnutrition and risk of malnutrition among older adults admitted to the hospital with a COVID-19 diagnosis is reported to be very high (52.7% and 27.5%) [15].

Due to the hypothesized greater susceptibility of older adults to COVID-19-related lifestyle changes and because of the potential detrimental consequences of these lifestyle changes for older adults’ health and functioning, research focusing specifically on older adults is warranted but very limited so far [16]. Therefore, the aims of the present explorative study were: (1) to investigate the self-reported impact of the COVID-19 pandemic on nutrition and physical activity behaviour in older participants of the Longitudinal Aging Study Amsterdam; (2) to investigate whether this impact is larger for those who were in quarantine for some time and (3) to identify subgroups of older adults who might be more susceptible for the negative impact of COVID-19 on nutrition and physical activity behaviour.

## 2. Methods

### 2.1. Study Population and Design

The Longitudinal Aging Study Amsterdam (LASA) is an ongoing cohort study that aims to explore the determinants, trajectories and consequences of physical, cognitive, emotional and social functioning in relation to aging in the Netherlands. LASA comprises three cohorts: the first cohort (aged 55–85 years at baseline) was recruited at LASA’s start in 1992–1993, the second one (55–65 years) in 2002–2003 and the third one (55–65 years) in 2012–2013. Follow-up data collection waves take place every 3–4 years. Details on the sampling and data collection procedures have been published and described elsewhere [17,18]. The LASA study was conducted according to the guidelines laid down in the Declaration of Helsinki and was approved by the ethical committee of the VU University Medical Centre (Amsterdam, the Netherlands). All participants provided written informed consent.

In 2020, a self-administered questionnaire regarding the impact of the COVID-19 pandemic was sent to 1485 eligible LASA participants. Eligibility was determined as having participated in the latest regular examination wave (2018–2019, *n* = 1701), being alive (*n* = 61 were excluded) and willing to participate in ancillary studies (*n* = 155 excluded). The questionnaire referred to the period between March 1, 2020 and the date of questionnaire completion, which ranged from 8 June to 8 October 2020. A total of 1128 participants completed the questionnaire (76% response rate) either on paper (*n* = 909), online (*n* = 198) or by telephone (*n* = 21). For the current analyses, the final analytic sample consists of 1119 participants who live at home independently.

### 2.2. COVID-19 Questionnaire

For this study the following information was obtained from the questionnaire: questions on the diagnosis of COVID-19, quarantine and hospitalisation because of COVID-19 and self-reported impact of the COVID-19 pandemic on nutrition and physical activity behaviour. Participants were asked whether a physician or health care professional told them they likely had COVID-19 (yes/no), whether they had been tested positive for the COVID-19 virus (yes/no) and whether they had been hospitalized because of COVID-19 (yes/no). Participants were also asked whether they had been in quarantine, either because a physician or health care professional told them to do so or based on their own decision. Questions were asked about the frequency of perceived changes in seven nutrition behaviours during the past weeks due to the COVID-19 pandemic. These behaviour changes included: Difficulty obtaining groceries, Skipping warm meals, Eating less than normal, Eating too little or losing weight, Snacking more, Drinking more alcoholic beverages and Gaining weight, with response options always, sometimes, never and don’t know. Similarly, the frequency of perceived changes in three physical activity behaviours was assessed, that is, Less physically active than normal, Less exercise than normal and Not enough physical activity or exercise.

### 2.3. Other Variables

Data on age, sex and region (the western, north-eastern or southern part of the Netherlands; the prevalence of COVID-19 infections during the data collection period was highest in the south) were derived from the municipal registries. Other data were obtained from the regular LASA 2018–2019 data collection wave. Data on household composition were used to determine whether the participants lived alone or with others. Body mass index (BMI, kg/m^2^) was calculated based on measured body weight and body height. BMI was categorised as: underweight (BMI < 22 kg/m^2^), normal weight (BMI 22 to 28 kg/m^2^), overweight (BMI ≥ 28 to 30 kg/m^2^) and obesity BMI ≥ 30 kg/m^2^ [19,20,21]. Physical activity in the past 2 weeks was obtained by using the LASA physical activity questionnaire (LAPAQ), which was validated against pedometer counts and activity diaries in older persons [22]. The average total time spent per day on at least moderate-intensity activities (walking outdoors, bicycling, heavy household activities and sports activities) was calculated and dichotomized according to the Dutch physical activity recommendation for older adults: <150 versus 150+ min/week [23]. Self-rated health was assessed with the question ‘How would you rate your current health?’ with five response options poor, sometimes good/sometimes bad, fair, good and excellent. The variable was categorised into poor to fair, good and excellent. The level of difficulty performing seven activities was asked to assess functional limitations: walk up and down a staircase of 15 steps without resting, use your own or public transportation, cut your own toenails, dress and undress yourself, sit down and stand up from a chair, walk outside during five minutes without stopping and take a shower or bath. The five response options were: no, I cannot; only with help, yes with much difficulty, yes with some difficulty, yes without help. A functional limitation was defined as having at least some difficulty. Functional limitations were categorised into no limitations, 1–2 limitations and 3–7 limitations.

### 2.4. Statistics

Descriptive results are presented using means with standard deviation or percentages. Differences in characteristics between those in (self) quarantine and not in (self) quarantine were tested with Student’s *t*-tests (for continuous variables) and Pearson’s Chi-square tests (for categorical variables, with a continuity correction for 2 × 2 tables). Differences in the frequency of nutrition and physical activity behaviour changes between those in (self) quarantine and those not in quarantine were tested similarly, with the response options ‘always’ and ‘sometimes’ combined as well as the response options ‘never’ and ‘don’t know’ combined to avoid cells with <5% observations. For two behavioural changes, Gaining weight and Less exercise than normal, all four response options could be used. Logistic regression analyses were used to test the associations between sample characteristics assessed before the COVID-19 pandemic (with the exception of age for which age on the date of questionnaire completion was used) as independent variables and reported impact on a nutrition or physical activity behaviour (dichotomized into reporting frequency ‘always’ or ‘sometimes’ versus never’ or ‘don’t know’) as the dependent variable. Results are presented as odds ratios with 95% confidence intervals. Data were analysed with SPSS version 26 and a *p*-value < 0.05 was considered statistically significant.

## 3. Results

Some characteristics of the total study sample, as well as stratified by quarantine (yes/no), are shown in Table 1. In the total sample, 28 participants reported to have been diagnosed with the COVID-19 infection by a health care professional and 1 participant reported to be positively tested. Three participants were admitted to the hospital because of COVID-19 infection complications. A total of 123 participants (11.5% of those who completed the quarantine questions) had been in quarantine during the COVID-19 pandemic. The median duration of the quarantine was 21 days (*N* = 106). Women and those with more functional limitations before the COVID-19 pandemic reported more often to have been in quarantine.

Table 2 shows the self-reported impact of the COVID-19 pandemic on nutrition and physical activity behaviour for the total sample, as well as stratified by having been in quarantine or not. Overall, the reported impact on physical activity behaviour was larger compared to nutrition behaviour. About half of the sample reported to be always (8%) or sometimes (41.3%) less physically active than normal. Regarding nutrition behaviour, always or sometimes Snacking more (32.4%) and Gaining weight (31.9%) were reported most often. In contrast, behaviour changes associated with undernutrition were also reported, such as having Difficulty obtaining groceries (15.1%), Skipping warm meals (9.1%), Eating less than normal (12.1%) and Eating too little or losing weight (6.9%) always or sometimes. For all behaviour changes, except two (Eating too little or losing weight and Drinking more alcoholic beverages), the reported impact was larger for those who had been in quarantine as compared to those who had not been in quarantine.

The association between the pre-COVID-19 characteristics of the total study sample and the reported impact of the pandemic on nutrition behaviour is shown in Table 3. Older age was associated with a higher risk of Eating too little or losing weight but a lower risk of Snacking more, Drinking more alcoholic beverages and Gaining weight. Women had a higher risk of Eating less than normal, Snacking more and Gaining weight. No associations were observed with region of living in the Netherlands. Living alone increased the risk of Skipping warm meals, Eating less than normal, Eating too little or losing weight and Snacking more. Those who were underweight were at greater risk of Eating too little or losing weight and at lower risk of Snacking more and Drinking more alcoholic beverages as compared to those with a normal body weight. In contrast, those overweight or obese had a higher risk of Gaining weight. A fair to poor self-rated health was associated with a higher risk of Difficulty obtaining groceries, Snacking more and Drinking more alcoholic beverages as compared to excellent self-rated health. Physical inactivity was associated with a higher risk of Snacking more and Drinking more alcoholic beverages. Finally, having functional limitations increased the risk of most nutrition behaviour changes two- to four-fold, with the exception of Drinking more alcoholic beverages and Gaining weight.

The association between pre-COVID-19 characteristics of the total study sample and the reported impact of the pandemic on physical activity behaviour is shown in Table 4. Older participants were less likely to report a negative impact of the pandemic on their physical activity or exercise levels than younger participants, while women were more likely to report a negative impact than men. Region of living, BMI, self-rated health and physical activity level was not associated with the pandemic impact on physical activity and exercise. Having functional limitations was associated with a negative impact on physical activity behaviour but not on exercise behaviour.

## 4. Discussion

This study is one of the first studies to explore the self-reported impact of the COVID-19 pandemic on nutrition and physical activity behaviour of older adults living independently. Older adults reported more frequently a negative impact on physical activity behaviours (48.3–54.3% of the sample) than on nutrition behaviours (6.0–32.4%). Older adults who had been in quarantine more frequently reported an impact on these lifestyle behaviours than those who had not been in quarantine. Finally, several pre-COVID-19 characteristics of the sample were identified to be associated with a greater likelihood to report a negative impact on these lifestyle behaviours.

With regard to the impact of the COVID-19 pandemic on nutrition behaviour, the impact was more frequently reported for behaviour changes contributing to overnutrition as compared to behaviour changes contributing to undernutrition. About 20–30% of the sample reported more snacking, more alcohol consumption and weight gain. This impact has also been reported for younger populations [3,5,6]. Staying at home more, differences in daily (work) routine, boredom, limited access to fresh healthy products, as well as pandemic-related stress and anxiety have been suggested as contributors to these unhealthy behaviour changes [3,6]. In our sample, women, those who were relatively younger, unhealthier, physically inactive and overweight before the pandemic were at greater risk to report nutrition behaviour changes that could lead to overnutrition. A previous study in younger adults also showed that being overweight or obese increased the risk of weight gain during the pandemic [3,6]. Furthermore, in a study conducted in a population aged 12 to 86 y, younger age was associated with an increase in appetite during the pandemic which can explain the observed weight gain [6].

About 10–15% of our study sample reported having difficulty obtaining groceries, skipping warm meals, eating less than normal and eating too little and losing weight, which could lead to undernutrition. This population subgroup was characterized by being relatively older, more likely to be female, living alone, being underweight (BMI < 22 kg/m^2^) and having functional limitations before the pandemic. Living alone was also shown to decrease the likelihood of increased food intake due to the pandemic in a younger sample (with 20% aged > 60 y) [4]. These results clearly show that the impact of the COVID-19 pandemic is not universal across older adults but depends on specific characteristics. Our results can help to identify those older adults who would benefit from specific nutrition attention in order to avoid under- and overnutrition as well as to avoid an aggravation of nutrition problems already present before the pandemic, such as being underweight or overweight.

About half of the sample reported to be less physically active during the COVID-19 pandemic than normal and to exercise less often. These results confirm previous studies conducted in predominantly younger adults [4,5] and older Japanese adults aged 65+ years [16]. In contrast to the latter study and other studies [8], the pre-COVID physical activity level in our study was not associated with the pandemic impact. Those not meeting the physical activity recommendation before the pandemic were as likely to report a decrease in both physical activity and exercise as those meeting the physical activity recommendation. This difference might potentially be explained by the fact that pre-COVID-19 physical activity levels in the study of Suzuki [16] were assessed retrospectively in contrast to our study, in which physical activity was actually assessed before the onset of the pandemic. Experiencing the COVID-19 pandemic may already have an impacted on the physical activity recall before the pandemic of study participants. In our study the impact of the pandemic was smallest for the oldest old, perhaps because they are generally less active than the young-old and relatively more of their activities take place in and around the house and could thus be continued during the pandemic. Striking is the strong negative impact of the pandemic on physical activity in those with functional limitations. This could indicate that they lacked (in)formal support to be physically active or exercise or were more likely to stay at home during the pandemic and are most vulnerable to the decline in physical activity.

A strength of this study is that it is one of the first studies investigating the impact of the COVID-19 pandemic on the lifestyle of older adults living independently. Moreover, we used a large and representative sample of older adults living across the Netherlands and had the availability of pre-COVID sample characteristics, which allowed the identification of subgroups more susceptible to the impact of the pandemic on lifestyle behaviour. Potential limitations are that we assessed the self-reported impact of the COVID-19 pandemic on lifestyle behaviour and did not objectively measure the actual lifestyle changes during the pandemic, such as repeated assessments of body weight, dietary intake and physical activity using accelerometery. Also, the impact of being diagnosed with a COVID-19 infection on lifestyle behaviour could not be studied as only few participants were infected. A final limitation is that the participants responding to the COVID-19 questionnaire (*n* = 1128) were less likely to live alone (28.7% versus 36.6%, *p* < 0.05) and less likely to report a fair to poor self-rated health (29.2% versus 37.6%, *p* < 0.05) before the COVID-19 pandemic as compared to those who were eligible but did not respond to the questionnaire (*n* = 357). This selection bias may have attenuated the associations observed. However, age, sex, region of living, number of functional limitations, percentage meeting the physical activity recommendation and BMI were similar between responders and non-responders.

## 5. Conclusions

In conclusion, the COVID-19 pandemic negatively impacts nutrition behaviour of many older adults, with some being more susceptible to eating less and losing weight (i.e., oldest-old, women, those living alone and those with a BMI < 22 kg/m^2^ or functional limitations before the pandemic) thereby increasing their risk for developing undernutrition and some more susceptible to increased snacking, more alcohol consumption and weight gain (i.e., young-old, women, those with poor to fair self-rated health and those physically inactive or overweight before the pandemic) thereby increasing their risk for overnutrition. Furthermore, a decline in physical activity and exercise due to COVID-19 pandemic was reported by almost 50% of older adults, with the young-old, women and those with functional limitations before the pandemic being most at risk for this decline. Older adults who had been in quarantine were more likely to report a negative impact on nutrition and physical activity behaviours compared to those not in quarantine. These results show that the COVID-19 pandemic may have induced unhealthy, self-perceived nutrition and physical activity behaviour changes in many older adults, which will likely accelerate their risk of developing malnutrition, frailty, sarcopenia and disability [24,25,26,27].

## Figures and Tables

**Table 1:** General characteristics of the Longitudinal Aging Study Amsterdam participants who completed the COVID-19 questionnaire and live independently (*n* = 1119).

	Total Sample	In (Self) Quarantine ^1^	Not in (Self) Quarantine	*p*-Value ^2^
		*n* = 123	*n* = 948	
Age,	*n* = 1119	*n* = 123	*n* = 948	0.506
mean (SD)	74 (7)	74 (8)	74 (7)	
Sex, %	*n* = 1119	*n* = 123	*n* = 948	0.012
Women	52.8	63.4	50.9	
Region of living, %	*n* = 1119	*n* = 123	*n* = 948	0.057
West	39.8	30.1	40.1	
North-east	29.7	29.3	30.4	
South	22.2	30.9	21.4	
Other	8.4	9.8	8.1	
Living situation, %	*n* = 1028	*n* = 118	*n* = 866	0.457
Living alone	28.7	31.4	27.6	
BMI category, %	*n* = 999	*n* = 118	*n* = 838	0.465
Underweight	8.6	5.9	8.8	
Normal weight	53.9	52.5	53.7	
Overweight	16.0	15.3	16.6	
Obesity	21.5	26.3	20.9	
Self-rated health, %	*n* = 1028	*n* = 118	*n* = 866	0.181
Excellent	14.6	10.2	15.2	
Good	56.3	55.1	56.6	
Fair to poor	29.1	34.7	28.2	
Physical activity, %	*n* = 1027	*n* = 118	*n* = 865	0.991
≥150 min/weeks	36.4	37.3	37.3	
Functional limitations, %	*n* = 1024	*n* = 117	*n* = 864	0.029
No limitations	43.4	35.9	44.2	
1–2 limitations	36.5	35.0	36.9	
3–7 limitations	20.1	29.1	18.9	

^1^ Quarantine data missing *n* = 48, ^2^ Difference between quarantine and no quarantine.

**Table 2:** Reported frequency of nutrition and physical activity behaviour changes in the past weeks due to the COVID-19 pandemic.

	Total Sample	In (Self) Quarantine ^1^	Not in (Self) Quarantine	*p*-Value ^2^
Difficulty obtaining groceries, %	*n* = 1072	*n* = 119	*n* = 909	0.009
Always	1.4	0.8	1.4	
Sometimes	13.7	22.7	12.5	
Never	83.8	72.3	85.3	
Don’t know	1.1	4.2	0.8	
Skipping warm meals, %	*n* = 1068	*n* = 116	*n* = 909	0.003
Always	0.3	0	0.3	
Sometimes	8.8	17.2	8.0	
Never	90.6	82.8	91.3	
Don’t know	0.3	0	0.3	
Eating less than normal, %	*n* = 1059	*n* = 115	*n* = 902	0.003
Always	0	0	0	
Sometimes	12.1	20.9	10.9	
Never	86.6	77.4	87.8	
Don’t know	1.3	1.7	1.3	
Eating too little or losing weight, %	*n* = 1075	*n* = 118	*n* = 914	0.260
Always	0.3	0	0.3	
Sometimes	6.3	9.3	5.8	
Never	86.2	88.1	90.6	
Don’t know	3.5	2.5	3.3	
Snacking more, %	*n* = 1083	*n* = 121	*n* = 918	<0.001
Always	3.4	9.1	2.8	
Sometimes	29.0	43.0	26.9	
Never	65.6	45.5	68.4	
Don’t know	2.0	2.5	1.9	
Drinking more alcoholic beverages, %	*n* = 1086	*n* = 119	*n* = 922	0.072
Always	0.7	2.5	0.5	
Sometimes	13.3	17.6	13.0	
Never	84.0	77.3	84.7	
Don’t know	2.0	2.5	1.7	
Gaining weight, %	*n* = 1081	*n* = 120	*n* = 917	0.021
Always	4.5	8.3	4.3	
Sometimes	27.4	35.0	26.4	
Never	63.6	51.7	65.1	
Don’t know	4.5	5.0	4.3	
Less physically active than normal, %	*n* = 1076	*n* = 119	*n* = 914	<0.001
Always	8.0	16.0	7.1	
Sometimes	41.3	52.1	39.8	
Never	49.4	31.1	51.8	
Don’t know	1.3	0.8	1.3	
Less exercise than normal, %	*n* = 1075	*n* = 116	*n* = 916	0.024
Always	16.7	22.4	15.9	
Sometimes	34.0	40.5	33.2	
Never	44.3	31.0	45.7	
Don’t know	5.1	6.0	5.1	
Not enough physical activity or exercise, %	*n* = 1084	*n* = 120	*n* = 921	0.005
Always	10.6	14.2	10.0	
Sometimes	43.7	52.5	42.8	
Never	43.0	31.7	44.4	
Don’t know	2.7	1.7	2.8	

^1^ Quarantine data missing *n* = 48, ^2^ Difference between quarantine and no quarantine.

**Table 3:** Association (OR with 95% CI) between pre-COVID-19 characteristics ^1^ of the total study sample and the reported impact ^2^ of the COVID-19 pandemic on seven nutrition behaviours.

	Difficulty Obtaining Groceries	Skipping Warm Meals	Eating Less than Normal	Eating too Little or Losing Weight	Snacking More	Drinking more Alcoholic Beverages	Gaining Weight
Determinants:							
Age, year	1.02 (0.99–1.05)	1.00 (0.97–1.03)	0.98 (0.95–1.01)	**1.04 (1.00–1.08)**	**0.95 (0.93–0.97)**	**0.95 (0.93–0.98)**	**0.94 (0.92–0.96)**
Sex (women vs. men)	1.51 (0.99–1.05)	1.57 (0.92–2.67)	**1.73 (1.08–2.78)**	0.80 (0.44–1.45)	**1.85 (1.35–2.53)**	0.82 (0.55–1.22)	**1.63 (1.19–2.23)**
Region							
West vs. other	1.37 (0.67–2.78)	1.38 (0.57–3.37)	1.41 (0.59–3.37)	0.77 (0.32–1.86)	0.97 (0.56–1.69)	0.92 (0.47–1.81)	0.97 (0.56–1.66)
North-east vs. other	0.90 (0.42–1.94)	0.73 (0.28–1.96)	0.90 (0.36–2.29)	0.59 (0.22–1.56)	0.91 (0.51–1.61)	0.89 (0.44–1.80)	0.70 (0.40–1.22)
South vs. other	0.90 (0.41–1.99)	1.50 (0.58–3.85)	2.20 (0.90–5.41)	0.92 (0.35–2.39)	0.97 (0.54–1.75)	0.79 (0.38–1.65)	0.61 (0.34–1.10)
Living situation							
(alone vs. with others)	1.30 (0.85–1.99)	**2.52 (1.51–4.20)**	**1.80 (1.12–2.87)**	**2.30 (1.28–4.13)**	**1.60 (1.15–2.24)**	1.07 (0.68–1.68)	1.12 (0.79–1.59)
BMI category							
Underweight vs. normal	1.57 (0.78–3.18)	1.06 (0.41–2.72)	0.80 (0.32–1.98)	**4.01 (1.70–9.45)**	**0.36 (0.19–0.68)**	**0.30 (0.11–0.86)**	0.20 (0.09–0.43)
Overweight vs. normal	0.80 (0.44–1.46)	1.30 (0.67–2.54)	1.53 (0.85–2.77)	1.04 (0.44–2.44)	1.43 (0.96–2.13)	1.60 (0.99–2.59)	**1.85 (1.24–2.75)**
Obesity vs. normal	1.45 (0.92–2.29)	1.14 (0.64–2.03)	1.46 (0.87–2.46)	1.88 (0.98–3.60)	1.27 (0.88–1.83)	1.12 (0.70–1.82)	**1.98 (1.37–2.86)**
Self-rated health							
Good vs. excellent	1.58 (0.75–3.35)	1.80 (0.68–4.78)	1.36 (0.64–2.90)	0.59 (0.26–1.35)	1.10 (0.71–1.70)	1.42 (0.78–2.59	0.87 (0.57–1.33)
Fair to poor vs. excellent	**2.52 (1.15–5.53)**	2.50 (0.90–6.92)	1.84 (0.81–4.14)	0.70 (0.29–1.72)	**1.76 (1.07–2.88)**	**2.20 (1.12–4.31)**	1.33 (0.82–2.18)
Physical activity							
(<150 vs. ≥150 min/weeks)	1.03 (0.68–1.57)	0.91 (0.55–1.52)	0.92 (0.58–1.45)	1.01 (0.56–1.83)	**1.45 (1.06–1.99)**	**1.63 (1.06–2.49)**	1.28 (0.93–1.75)
Functional limitations							
1–2 vs. 0 limitations	**2.88 (1.70–4.88)**	**2.53 (1.31–4.88)**	**1.76 (1.02–3.06)**	**3.48 (1.61–7.50)**	**1.79 (1.28–2.52)**	1.10 (0.72–1.68)	1.20 (0.85–1.68)
3–7 vs. 0 limitations	**4.14 (2.21–7.78)**	**3.81 (1.74–8.34)**	**2.98 (1.52–5.85)**	**3.65 (1.43–9.32)**	**1.71 (1.07–2.73)**	0.66 (0.35–1.24)	0.97 (0.60–1.57)

OR = odds ratio, 95% CI = 95% confidence interval. Statistically significant associations are indicted in bold. ^1^ Except for age: age during the COVID-19 questionnaire completion was used. ^2^ Including impact frequency of ‘always’ and ‘sometimes.’

**Table 4:** Association (OR with 95% CI) between pre-COVID-19 characteristics ^1^ of the total study sample and the reported impact ^2^ of the pandemic on three physical activity behaviours.

	Less Physically Active than Normal	Less Exercise than Normal	Not Enough Physical Activity or Exercise
Determinants:			
Age, y	**0.97 (0.95–0.99)**	**0.97 (0.95–0.99)**	**0.96 (0.94–0.98)**
Sex (women vs. men)	**1.47 (1.10–1.96)**	**1.54 (1.16–2.05)**	**1.49 (1.12–1.98)**
Region			
West vs. other	1.15 (0.70–1.91)	0.97 (0.59–1.59)	1.31 (0.80–2.15)
North-east vs. other	0.94 (0.56–1.58)	0.95 (0.57–1.59)	1.03 (0.62–1.72)
South vs. other	1.20 (0.70–2.05)	1.15 (0.68–1.94)	1.22 (0.72–2.07)
Living situation			
(alone vs. with others)	**1.47 (1.07–2.02)**	1.10 (0.80–1.50)	1.35 (0.98–1.86)
BMI category			
Underweight vs. normal	0.70 (0.43–1.15)	0.85 (0.53–1.37)	0.66 (0.41–1.06)
Overweight vs. normal	0.83 (0.57–1.22)	1.06 (0.73–1.55)	0.95 (0.65–1.38)
Obesity vs. normal	0.99 (0.70–1.41)	0.91 (0.64–1.29)	0.93 (0.65–1.32)
Self-rated health			
Good vs. excellent	1.15 (0.78–1.69)	1.18 (0.81–1.72)	1.29 (0.88–1.88)
Fair to poor vs. excellent	1.33 (0.85–2.10)	1.23 (0.79–1.92)	1.56 (0.99–2.45)
Physical activity			
(<150 vs. ≥150 min/wk)	1.26 (0.95–1.68)	0.93 (0.70–1.23)	1.21 (0.91–1.61)
Functional limitations			
1–2 vs. 0 limitations	**1.58 (1.16–2.15)**	1.02 (0.75–1.39)	**1.41 (1.04–1.92)**
3–7 vs. 0 limitations	**2.46 (1.57–3.85)**	0.96 (0.62–1.48)	**1.82 (1.17–2.84)**

OR = odds ratio, 95% CI = 95% confidence interval. Statistically significant associations are indicted in bold. ^1^ Except for age: age during the COVID-19 questionnaire completion was used. ^2^ Including impact frequency of ‘always’ and ‘sometimes’.
